# The *MMP-1*, *MMP-2*, and *MMP-9* gene polymorphisms and susceptibility to bladder cancer: a meta-analysis

**DOI:** 10.1007/s13277-013-1395-6

**Published:** 2014-01-05

**Authors:** Yulan Yan, Hongjie Liang, Taijie Li, Meng Li, Ruolin Li, Xue Qin, Shan Li

**Affiliations:** Department of Clinical Laboratory, First Affiliated Hospital of Guangxi Medical University, 6 Shuangyong Road, Nanning, 530021 Guangxi People’s Republic of China

**Keywords:** Bladder cancer, *MMP*, Polymorphism, Meta-analysis

## Abstract

The relationship between matrix metalloproteinase (*MMP*) polymorphisms and bladder cancer risk has become a hot topic and was studied extensively in recent years, but the results are still controversial. In order to estimate the relationship of *MMP* polymorphisms and the risk of bladder cancer, we performed this meta-analysis. We conducted a comprehensive search of databases; PubMed, Web of Science, Embase, Chinese Biomedical Literature Database (CBM, Chinese) and Wanfang Database (Chinese) were searched for all case–control studies which mainly study the relationship between *MMP-1*-1607 1G/2G, *MMP-2*-1306 C/T, and *MMP-9*-1562 C/T polymorphisms and the susceptibility of bladder cancer. The association between the *MMP* polymorphisms and bladder cancer risk was conducted by odds ratios (ORs) and 95 % confidence intervals (95 % CIs). At last, totally five literatures with 1,141 cases and 1,069 controls were contained in the meta-analysis. Among these articles, four articles with 1,103 cases and 1,053 controls were about *MMP-1*-1607 1G/2G polymorphism and three studies with 839 cases and 775 controls for *MMP-2*-1306 C/T polymorphism and *MMP-9*-1562 C/T polymorphism. With regard to *MMP-1*-1607 1G/2G polymorphism, significant association was found with bladder cancer susceptibility only under recessive model (2G2G vs. 1G2G/1G1G: OR = 1.44, 95 % CI = 1.05–1.97, *P* = 0.022), and as to the *MMP-2*-1306 C/T polymorphism, significant association was found with bladder cancer susceptibility only under homozygote model (TT vs. CC: OR = 2.10, 95 % CI = 1.38–3.10, *P* = 0), but no associations was found between *MMP-9*-1562 C/T polymorphism and bladder cancer susceptibility. The results suggest that the *MMP-2*-1306 C/T and *MMP-9*-1562 C/T polymorphisms are significantly associated with bladder cancer susceptibility, and no associations were found between *MMP-9*-1562 C/T polymorphism and bladder cancer susceptibility.

## Introduction

Bladder cancer is one of the most common malignant diseases around the world that has the highest recurrence rate of any malignancy [1–3]. The most common type of bladder cancer is transitional cell carcinomas, and the remainders are squamous tumors, adenocarcinomas, and other subtypes [4]. Its occurrence increases with age, and it is greater in men. Although there have been increasingly intensive researches on bladder cancer over the past several decades, there are little advances in the understanding of the pathogenesis of bladder cancer [2, 5]. Previous studies have reported that several environmental factors may be considered as the risk to bladder cancer, for instance of smoking, chronic inflammation, anticancer drugs, aromatic amines contained in dyes, radiation exposure, and so on [6, 7]. However, many people exposed to these risk factors do not develop bladder cancer, while among those people not exposed to the risk factors listed above, a large number of patients develop bladder cancer, and this suggests that in addition to environmental factors, genetic factors may play an important role in the development of bladder cancer [8, 9]. Host factors, such as genetic polymorphisms, have been reported as a risk factor in the development of cancers; bladder cancer is one of the cancers [10–12].

Previous studies have reported that genetic variants in genes encoding proteins like matrix metalloproteinase (*MMP*) enzymes may contribute to the development of bladder cancer [13, 14]. *MMP* is a family of matrix metalloproteinases and a zinc-dependent endopeptidase enzyme that can reduce substantially all of the extracellular matrix components such as basement membrane, collagen, and fibronectin [15–17]. *MMP*s play an important role not only in physiological but also in pathological conditions, including tissue regeneration, wound repair, reproduction, arthritis, atherosclerosis, and so on [18]. The *MMP-1*, *MMP-2*, and *MMP-9* genes are three important members of the *MMP* family. The polymorphisms of *MMP-1*-1607 1G/2G, *MMP-2*-1306 C/T, and *MMP-9*-1562 C/T were reported to be related with bladder cancer susceptibility, but the conclusions remain to be inconsistent [13, 19–23]. So, to get a more accurate result, we conducted this meta-analysis.

## Materials and methods

### Publication search

We conducted a comprehensive search strategy through searching the electronic databases, such as the PubMed, Web of Science, Embase, Chinese Biomedical Literature Database (CBM, Chinese), and Wanfang Database (Chinese) using the following search terms: *MMP*, “matrix metalloproteinase,” and “collagenase” combined with “bladder cancer,” “bladder carcinoma,” and “polymorphism,” “single nucleotide polymorphism (SNP),” and “variation” for all study publications before July 2013, and there was no language restriction in the literature search. Additional studies were identified by a hand search of the references of original research, and reviews were also examined in order to find more eligible studies. With regard to published studies of the same author, with overlapping data, we selected the most recent or complete study only.

### Inclusion and exclusion criteria

The inclusion criteria of our meta-analysis were as follows: (a) evaluation of the *MMP-1*-1607 1G/2G, *MMP-2*-1306 C/T, and *MMP-9*-1562 C/T polymorphism and bladder cancer risk; (b) case–control study; (c) provided sufficient genotype data in order to calculate the odds ratio (OR) with 95 % confidence interval; (d) genotype frequencies in controls was abided by Hardy–Weinberg equilibrium (HWE). The following were exclusion criteria: (a) not a case–control study, (b) reviews or case reports, (c) no available data reported, and (d) duplicated reports.

### Data extraction

Two of our authors independently extract the information from each research report according to the inclusion criteria listed above. When they have different opinions, they would reach agreement through discussion. If they cannot reach consensus, the third author would consulted to resolve the contradiction. The following information were extracted: (a) first author's name, (b) publication years, (c) country of origin, (d) ethnicity, (e) genotyping methods, (f) source of the control group, (g) sample size of cases and controls, and (h) type of genotype. We also evaluated whether the genotype distributions followed the Hardy–Weinberg equilibrium.

### Statistical analysis

The possible association between the *MMP-1*-1607 1G/2G polymorphism with the risk of bladder cancer was evaluated by OR and 95 % confidence interval (CI) according to allele contrast (2G vs. 1G), homozygote (2G2G vs. 1G1G), heterozygote (1G2G vs. 1G1G), recessive (2G2G vs. 1G2G/1G1G), and dominant (2G2G/1G2G vs. 1G1G) models, while the strength of association between the *MMP-2*-1306 C/T and *MMP-9*-1562 C/T polymorphisms and bladder cancer susceptibility was evaluated by OR and 95 % CI according to allele contrast (T vs. C), homozygote (TT vs. CC), heterozygote (TC vs. CC), recessive (TT vs. TC/CC), and dominant (TT/TC vs. CC) models, respectively. The heterogeneity was tested by a chi-square-based *Q* statistic test. The effect of heterogeneity was quantified by using *I* ^2^ value as well as *P* value [24]. If *I* ^2^ value is >50 % or *P* < 0.10, suggesting that an obvious heterogeneity existed, ORs were pooled by random effect model [25]. Otherwise, the fixed effect model was used [26].

We assessed the HWE for the control group in every article by using the professional web-based program (http://ihg2.helmholtz-muenchen.de/cgibin/hw/hwa1.pl), if *P* > 0.05 suggests that the controls followed HWE balance. A sensitivity analysis was carried out to assess whether there is stability of our results. By way of deleting a single study, there was a time to assess whether the study results have an impact on the overall pooled ORs. [27]. Publication bias was assessed using Egger’s test (*P* < 0.05 indicates that statistically significant publication bias existed) [28] and visual observation of the funnel plot [29]. All statistical tests were conducted using the Stata Software (version 9.2, Stata Corp).

## Results

### Search results and study characteristics

A total of five articles [13, 19–23] with 1,141 cases and 1,069 controls were contained in this study after a careful examination based on the inclusion criteria above, four studies with 1,103 cases and 1,053 controls for *MMP-1*-1607 1G/2G polymorphism, three studies with 839 cases and 775 controls for *MMP-2*-1306 C/T polymorphism, and three studies with 839 cases and 775 controls for *MMP-9*-1562 C/T polymorphism; the general characteristics of studies included in the meta-analysis were listed in Table 1. The genotype distributions in the controls of all studies were consistent with HWE.

**Table 1:** General characteristics of studies included in the meta-analysis

First author	Year	Country	Ethnicity	Method of genotyping	Source of control	Sample size (case/control)	Type of genotype
Srivastava [19]	2010	Indian	Asian	PCR-RFLP	PB	200/200	MMP-1-1607
Kader [21]	2006	USA	Caucasian	Taqman	HB	560/560	MMP-1-1607
							MMP-2-1306
							MMP-9-1562
Tasci [22]	2008	Turkey	Caucasian	PCR-RFLP	PB	102/94	MMP-1-1607
Wieczorek [13]	2013	Poland	Caucasian	Taqman	PB	241/199	MMP-1-1607
							MMP-2-1306
							MMP-9-1562
Zhong [23]	2005	China	Asian	PCR-RFLP	HB	38/16	MMP-1-1607
							MMP-2-1306
							MMP-9-1562

*PCR–RFLP* PCR–restriction fragment length polymorphism, *HB* hospital based, *PB* population based

### Meta-analysis results

**Fig. 1:**
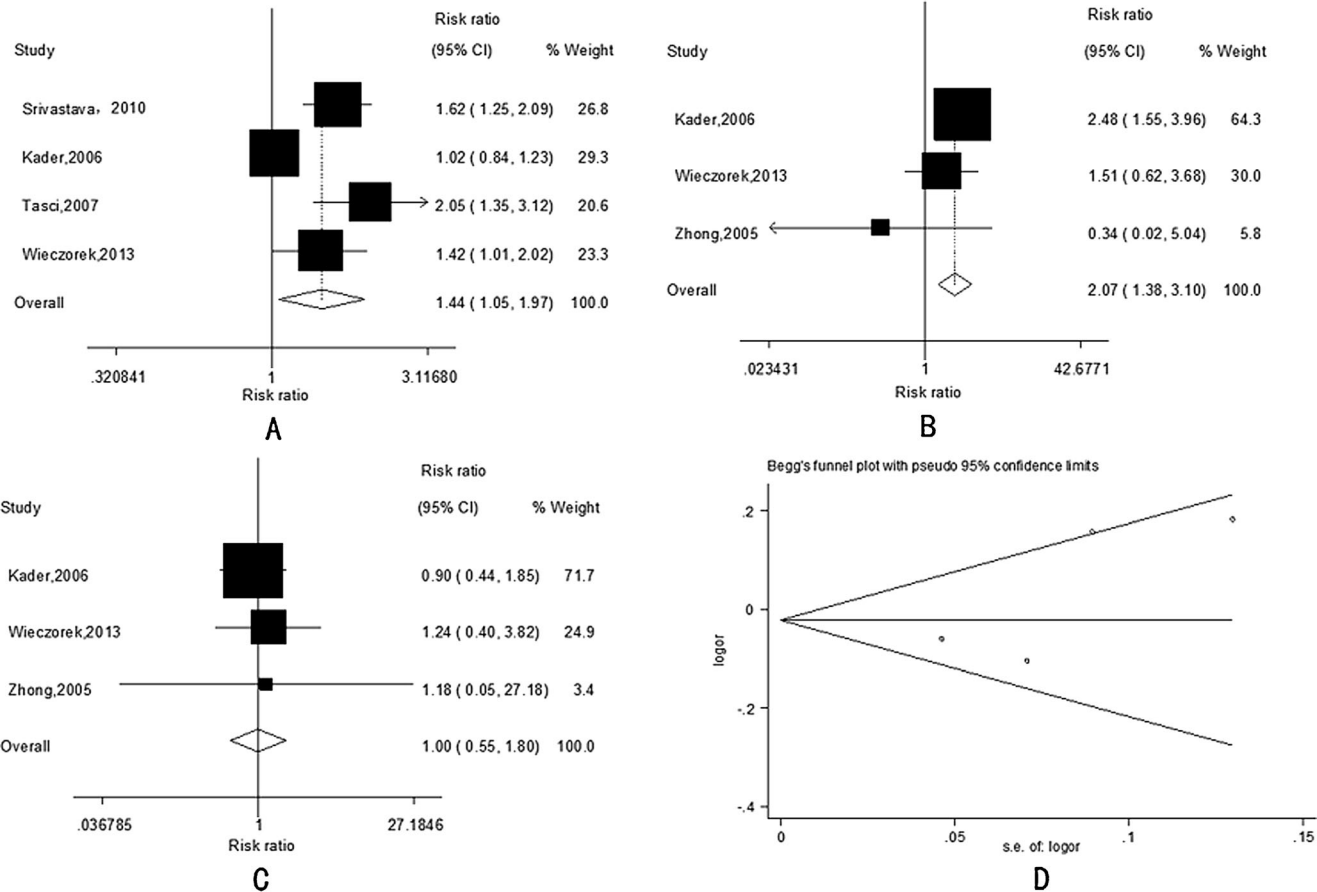
**a** The forest plot describing the meta-analysis under recessive model for the association between *MMP-1*-1607 1G/2G polymorphism and the risk of bladder cancer (2G2G vs. 1G2G/1G1G). **b** The forest plot describing the meta-analysis under homozygous model for the association between *MMP-2*-1306 C/T polymorphism and the risk of bladder cancer (TT vs. CC). **c** The forest plot describing the meta-analysis under homozygous model for the association between *MMP-9*-1562 C/T polymorphism and the risk of bladder cancer in Chinese population (TT vs. CC). **d** Begg funnel plot for publication bias test for the association between *MMP-1* polymorphism and the risk of bladder cancer. *Each point* represents a separate study for the indicated association. *Log (OR)* natural logarithm of OR. *Horizontal line* means the effect size

The main results of this meta-analysis and the heterogeneity assessment were shown in Tables 2, 3, and 4. With regard to *MMP-1*-1607 1G/2G polymorphism, significant association was found with bladder cancer risk only under recessive model (2G2G vs. 1G2G/1G1G: OR = 1.44, 95 % CI = 1.05–1.97, *P* = 0.022, Fig. 1a), and as to *MMP-2*-1306 C/T polymorphism, significant association was found with bladder cancer risk only under homozygote model (TT vs. CC: OR = 2.10, 95 % CI = 1.38–3.10, *P* = 0, Fig. 1b), but we did not find any associations between *MMP-9*-1562 C/T polymorphism and bladder cancer risk (which only show homozygote model (TT vs. CC) in Fig. 1c).

**Table 2:** Results of meta-analysis for MMP-1-1607 1G/2G polymorphism and bladder cancer risk

Comparison	Test of association			Model	Test of heterogeneity	
	OR	95 % CI	*P*		*P*	*I* ^2^
2G vs. 1G	1.17	0.98–1.39	0.083	R	0	88.2
2G2G vs. 1G1G	1.26	0.95–1.67	0.103	R	0	83.8
1G2G vs. 1G1G	1.02	0.89–1.16	0.799	R	0.035	65.0
2G2G vs. 1G2G/1G1G	1.44	1.05–1.97	0.022	R	0.003	79.0
2G2G/1G2G vs. 1G1G	1.06	0.95–1.20	0.307	R	0.001	82.8

*OR* odds ratio, *CI* confidence interval, *F* fixed effect model, *R* random effect model

**Table 3:** Results of meta-analysis for MMP-2-1306 C/T polymorphism and bladder cancer risk

Comparison	Test of association			Model	Test of heterogeneity	
	OR	95 % CI	*P*		*P*	*I* ^2^
T vs. C	1.13	0.71–1.81	0.599	R	0.001	86.4
TT vs. CC	2.10	1.38–3.10	0	F	0.251	27.8
TC vs. CC	1.12	0.64–1.95	0.698	R	0	90.6
TT vs. TC/CC	1.42	0.93–2.16	0.110	F	0.669	0
TT/TC vs. CC	1.11	0.68–1.83	0.679	R	0	90.1

*OR* odds ratio, *CI* confidence interval, *F* fixed effect model, *R* random effect model

**Table 4:** Results of meta-analysis for MMP-9-1562 C/T polymorphism and bladder cancer risk

Comparison	Test of association			Model	Test of heterogeneity	
	OR	95 % CI	*P*		*P*	*I* ^2^
T vs. C	0.98	0.83–1.15	0.787	F	0.347	5.6
TT vs. CC	1.00	0.55–1.80	0.998	F	0.891	0
TC vs. CC	0.97	0.82–1.15	0.719	F	0.270	23.6
TT vs. TC/CC	1.00	0.55–1.82	0.994	F	0.941	0
TT/TC vs. CC	0.97	0.83–1.14	0.740	F	0.280	21.4

*OR* odds ratio, *CI* confidence interval, *F* fixed effect model, *R* random effect model

### Sensitive analysis and publication bias

To estimate the sensitivity of our meta-analysis, a leave-one-out sensitivity analysis was performed. A single article included in the meta-analysis was assessed each time to reflect the impact of the individual data set to pooled ORs. Any single study was omitted, while the overall statistical significance does not change, indicating that the results are stable (data not shown). Therefore, the sensitivity analysis results show that our meta-analysis data is relatively stable and credible.

Both funnel plot and Egger’s test were conducted to access the publication bias. Funnel plot is relatively straightforward to observe whether the publication bias is presence, and Egger’s test was used to provide a statistical evidence of funnel symmetry. Both the shapes of the funnel plot (Fig. 1d) and Egger’s test (all *P* > 0.05, data not shown) suggest that no publication bias existed in our meta-analysis.

## Discussion

Bladder cancer is one of the most common urinary malignant diseases around the world [2]. However, in the incidence of bladder cancer, the mechanism is not currently clear [5]. There is growing evidence for the important roles of genetic factors in the host’s susceptibility to bladder cancer [30–32]. Currently, there are several genetic polymorphisms which have been identified as risk factors of bladder cancer, such as cyclin D1 (CCND1) G870A polymorphism [10] and NQO1 C609T polymorphism [33]. There were a series of studies that have looked into the association between the *MMP* polymorphisms and bladder cancer susceptibility; however, the results obtained were inconsistent or controversial [13, 19–23].

Meta-analysis is a powerful tool that combines the world’s research literature that can resolve the statistical power and discrepancy problem in associated studies [34]. It is a more systematic statistical method than any single case–control studies or cohort studies [35, 36], and it may investigate a large number of individuals, and so, the risk of disease can be estimated with the impact of a genetic factor on disease susceptibility [37]. In the current study, a total of five case–control studies with 1,141 cases and 1,069 controls were included in the meta-analysis [13, 19–23], and the association between *MMP-1*-1607 1G/2G, *MMP-2*-1306 C/T, and *MMP-9*-1562 C/T polymorphisms and bladder cancer risk was explored. Our results suggest that *MMP-1*-1607 1G/2G polymorphism was significantly associated with bladder cancer risk under recessive model, and significant association was found between *MMP-2*-1306 C/T polymorphism and bladder cancer risk under homozygote model, but there is no association found between *MMP-9*-1562 C/T polymorphism and bladder cancer risk. The results indicate that potentially functional *MMP-1*-1607 1G/2G and *MMP-2*-1306 C/T polymorphisms may play an important role in the development of bladder cancer.

In spite of comprehensive analysis conducted to show the association between *MMP* polymorphisms and bladder cancer risk, there are still some limitations that should be pointed out. Firstly, the sample size of each study and the quantity of studies included in our meta-analysis were relatively small. The total sample size may not be enough to make a convincing conclusion. Secondly, there is no uniform definition of the control group. Some studies used population-based controls, while others used hospital-based controls, which may not be a representative of the general population. Thirdly, only published studies were included in our meta-analysis, which is likely to miss some relevant unpublished but may meet the inclusion criteria articles. Thus, publication bias may be incurred. Fourthly, the possible interactions of gene–environment and gene–gene were not assessed in the meta-analysis.

Therefore, larger-scale and well-designed studies are necessary to estimate the association between MMP polymorphisms and the risk of bladder cancer in the future. Besides, the possible gene–environment and gene–gene interactions should be considered in the future studies too.

In conclusion, the results of our meta-analysis suggest that a significant association was found between the MMP-2-1306 C/T and MMP-9-1562 C/T polymorphisms and the risk of bladder cancer, while the *MMP-9*-1562 C/T polymorphism is not associated with bladder cancer. Considering the limitations listed above, larger-scale and well-designed studies are require to further estimate the association between *MMP* polymorphisms and bladder cancer risk in future studies. Besides, the possible gene–environment and gene–gene interactions should also be considered in future meta-analysis.
